# The Molecular Basis of Partial Reversal or Significant Slowing of ALS, Parkinson’s Disease, and Lewy Body Dementia by Mesenchymal Exosomes/Secretome

**DOI:** 10.3390/ijms27104483

**Published:** 2026-05-16

**Authors:** Chadwick C. Prodromos, Ruby Del Villar, Andrew Striegel, Gerard Pena, Rohan Dixit

**Affiliations:** Prodromos Stem Cell Institute, 1714 Milwaukee Ave, Glenview, IL 60025, USA; rubyd@thepsci.com (R.D.V.); andrews@ismoc.net (A.S.); gerardp@thepsci.com (G.P.); rohand@thepsci.com (R.D.)

**Keywords:** exosome, secretome, ALS, LBD, PD

## Abstract

Neuromuscular and neurodegenerative (NMND) disorders are diseases that cause progressive damage to the central nervous system leaving patients with symptoms that negatively affect everyday living with death almost inevitable. These include amyotrophic lateral sclerosis (ALS), Lewy body dementia (LBD), and Parkinson’s disease (PD) with cases expected to increase in the future. Intranasally administered stem cell-derived exosomes/secretome have been seen as potential therapeutic options for these disorders in preclinical animal models. This study sought to observe the efficacy of mesenchymal stem cell-derived exosomes/secretome in patients with ALS, LBD, and PD. Based off these preclinical studies, we conducted a case-controlled series experiment with 86 patients with ALS, LBD, or PD, with the independent variable being the treatment and the dependent variable being the clinical response. These patients were recruited and given intranasal instillations of various MSC-derived exosome/secretome products. Subsequent treatments were given to patients who did not have a response to one product. Patients were followed up at one week, one, two, three, and six months post-treatment. Historical external controls were used for comparison to clinical outcomes. There were no serious adverse events in any patient. A total of 67 of 86 (77%) patients showed a positive clinical response to at least one product. Outcomes were strongly associated with greater treatment frequency for ALS and LBD. Intranasal administration of MSC-derived exosome/secretome products were safe, and most patients showed overall improvement with at least one product. Some patients also saw a substantial decrease in the rate of decline compared to historical controls. These results also give rise to the hypothesis: do MSC-derived exosomes/secretome treatments show efficacy in other NMND disorders? The primary limitation of this study is the 6-month follow-up.

## 1. Introduction

Neuromuscular and neurodegenerative (NMND) disorders such as amyotrophic lateral sclerosis (ALS), Lewy body dementia (LBD), and Parkinson’s disease (PD) are among the most feared human afflictions due to their severe and relentless debilitating effects [1,2,3,4]. There are more than 57 million people worldwide who suffer from neurodegenerative disorders, and that number is anticipated to double every 20 years [5].

ALS is a rare disease characterized by dysfunction in the upper and lower motor neurons causing weakening of limb movement, dysphagia, and respiratory issues [1]. In addition to a decline in cognition, all these symptoms worsen over time leading to the patient becoming immobile and inevitably dying, with death usually occurring 24 to 50 months after symptom onset [6].

PD is the second most common neurodegenerative disease characterized by bradykinesia, tremors, rigidity, and deviations in the gait cycle causing movement issues [3]. Besides hindering movement, PD also causes other symptoms such as urinary incontinence, memory loss, cognitive decline, pain, and insomnia [3]. There are more than 6 million people who have PD with prevalence rates expected to increase [3].

LBD is a disease that consists of two clinical diagnoses, Parkinson’s disease dementia (PDD) and dementia with Lewy bodies (DLB) [2]. It is the second most common cause of neurodegenerative dementia after Alzheimer’s with around 1.4 million Americans who live with it [2]. LBD is characterized by episodic memory impairment, cognitive fluctuations, issues with perception, and PD-like movement problems such as slow gait, posture instability, bradykinesia, and rigidity [2].

There is no effective treatment for ALS or LBD [1,7], and PD has no disease-modifying treatments [8]. Disease-modifying treatments exist for some forms of MS, but they carry risks of severe side effects, and some treatments only have marginal benefits [9]. With no cures on the horizon and the expected influx in NMND cases, the need for a disease-modifying treatment that can be used for all NMND disorders is drastically increasing. Intravenous mesenchymal stem cells (MSCs) have been seen as a candidate to treat NMND disorders showing promising results in animal models, but it has shown no remarkable efficacy in humans [10].

An alternative, emerging treatment option for NMND disorders is exosome and secretome treatment. Exosomes are small vesicles that are 40–200 nm in diameter and are secreted by cells; they contain an array of proteins, lipids, and nucleic acids that give exosomes the ability to facilitate intracellular communication and maintain cellular homeostasis [11]. The secretome is classified as the collective secretions from cells which contain proteins, free nucleic acids, lipids, and extracellular vesicles (EVs) including exosomes [12]. Researchers have specifically looked at stem cell-derived exosomes/secretome as a potential therapeutic in diseases and regenerative medicine [12]. There are numerous preclinical studies in animal models where intranasally and/or intravenously instilled exosomes/secretome from stem cells for ALS and PD had complete safety and consistent efficacy [13,14,15,16,17,18,19,20,21,22,23,24,25,26,27,28,29,30,31,32,33]. From this prior data, we hypothesized that intranasal MSC-derived exosome or secretome solutions would consistently produce a significant improvement in the natural history of ALS, PD, and LBD in humans relative to historical, external, natural history, and treatment controls that would exceed benefits from all other established treatment options for these diseases; failure in this study would be considered as absent or minimal nonsignificant regression in the progression of the disease seen in the majority of the cohort. In addition to our hypothesis, our objective is to demonstrate that MSC-derived exosomes and secretome show positive efficacy in NMNDs in humans with our primary question being whether the clinical outcomes affirm or refute our hypothesis.

## 2. Results

A total of 41/51 ALS, 20/29 PD, and 6/6 LBD patients had a meaningful clinical response to at least one product. Roughly 80% of ALS and PD patients responded to one treatment type or the other. Subsequent treatments were carried out with whichever treatment proved effective. There were no adverse events of any kind in any patient. Greater frequency of treatment was associated with improved results in ALS and LBD patients. ALS patients often experienced improved speech, swallowing, breathing and motor function, while many patients with PD had reduced mask-like facies, pain from rigidity, tremors and mental clouding, while experiencing improved balance, taste and smell. Some patients also reported having better sleep the night after treatment. A total of 11 patients with ALS received monthly treatments, and 11 additional patients received treatments every 2 months. The disease has been largely arrested in nine patients who are treated monthly, and overall improvements were seen in four of these patients. Among the bimonthly patients, eight patients have had the disease largely arrested, four of whom have shown overall improvement. Average Amyotrophic Lateral Sclerosis Functional Rating Scale Revised (ALSFRS-R) scores at each follow-up are shown in Table 1. It should be noted that sample sizes for each time point vary since sample size is a function of our ability to recruit patients to obtain follow-up.

Though no historical controls exist for LBD, they do exist for ALS and PD. An analysis of the PRO-ACT ALS natural history database by Bedlack et al. has shown that approximately 25% of patients over a 6-month interval do not decline [34]. In our study, 30% of patients had a plateau in disease progression lasting at least 6 months, while another 35% of patients had an improved ALSFRS-R score relative to baseline lasting at least 6 months.

The average slope of the ALSFRS-R was +0.20 points/month (SD 0.66) in patients with 3-month follow-up, and −0.14 points/month (SD 0.69) in patients with 6-month follow-up. Compared to average rate of decline in the PRO-ACT registry of −1.02 points/month (SD 2.3) [35], our patients had a significantly better rate of change at 3 months (*p* = 0.005, 95% CI [0.3678, 2.0722]), but no difference was observed at 6 months (*p* = 0.23, 95% CI [−0.5459, 2.3059]) due to the small sample size.

One PD study examined the efficacy and safety of rasagiline as an adjunctive therapy for Japanese PD patients with stable levodopa use [36]. During the two-week run-in period, patients continued to receive levodopa at their normal dosage, before being randomly assigned to receive rasagiline (0.5 mg or 1 mg) or placebo for 26 weeks. The change in Parkinson’s Disease Questionnaire 39 (PDQ-39) summary index between baseline and week 26 was measured. The placebo group (n = 141) had a least squares (LS) mean of +2.84 (SD = 9.61), the 0.5 mg rasagiline group (n = 134) had a LS mean of +0.33 (SD = 9.60), and the 1 mg rasagiline group (n = 129) had a LS mean of −1.0 (SD = 9.73). In our study, of patients with 6-month follow-up, we found the average monthly change in PDQ-39 to be −0.49 points/month (SD = 1.65), which is better than the placebo and 0.5 mg rasagiline groups in the Japanese study. It is important to note that compared to the Japanese placebo group, our patients showed no statistically significant difference (*p* = 0.27, 95% CI [−2.699, 9.3594]) due to the small sample size, and this was also seen in the 0.5 mg rasagiline group (*p* = 0.78, 95% CI [−5.2067, 6.8467]). Nonetheless, our patients’ PDQ-39 rate of change at 6 months is still better than those external controls.

## 3. Discussion

The main finding of this study was that intranasal exosome/secretome instillation in humans was completely safe and consistently effective in treating ALS, PD, and LBD as shown by 77% of patients showing clinical benefits from at least one product and achieving lower rate of declines in ALS and PD compared to external controls.

### 3.1. Intranasal Exosomes vs. Intravenous Stem Cells and How/Why Exosomes Work

There are two possible reasons for this intranasal exosome efficacy while intravenous stem cell treatment has been found to be ineffective. The first and most likely reason is that greater brain neuronal cellular effects are achieved from the intranasal route in comparison to levels achieved from the intravenous route which is subject to the blood–brain barrier. The blood–brain barrier (BBB) refers to tight junctions around brain capillaries which limit outward diffusion from these capillaries into brain neurons [37], making it difficult for particles to enter the brain cells if using the intravenous route. In contrast, the intranasal route of administration has been used by the pharmaceutical industry and pain specialists for introduction of drugs into the brain with success for decades [38,39].

#### 3.1.1. Molecular Routes of Exosomes

Two major proposed routes of intranasally administered drugs, exosomes, or biologics is that they migrate upward along the olfactory nerve or trigeminal nerve, with the olfactory nerve directly connected to the brain and the trigeminal nerve connected to the brainstem [40]. To access the nasal cavity, the olfactory nerve pierces through the cribriform plate while the trigeminal nerve innervates the nasal cavity which allows bypass of the BBB and direct access to the brain and brainstem [41,42]. These two proposed transport routes are supported by multiple preclinical biodistribution studies. Though not exosome-focused, an early biodistribution study compared the intranasal and intravenous routes of insulin-like growth factor-1 (IGF-1) in adult rats, and Thorne et al. found through high resolution phosphor imaging that intranasal IGF-1 accumulated in both the olfactory bulb and trigeminal nerve with concentrations of ~3.43 nM and ~36.4 nM respectively, with more IGF-1 also found in the midbrain, cerebellum, medulla, cervical spinal cord, thoracic spinal cord and other regions in the brain, supporting the mechanisms of these two pathways [40]. These concentrations were significantly higher when given intranasally, in contrast to the intravenous route where the IGF-1 concentrations in the brain and spinal cord were miniscule [40]. A more recent study looked at the intranasal administration of copper-labeled exosomes (1.2 × 10^10^) in rats, and autoradiography scans further confirmed the presence of exosomes in the olfactory nerve, cerebrum, brainstem, and trigeminal nerve with average particle counts of ~100,000, ~9000, ~15,000, and ~9500 respectively [43].

Alternatively, it is also possible that intranasally administered exosomes take other non-neuronal routes to access the brain from the nasal mucosa. These proposed paracellular and transcellular pathways all start with absorption through the olfactory epithelium, but pathways vary once leaving the epithelium into the nasal lamina propria [44]. The two possible pathways to the brain are absorption into blood vessels in the lamina propria to have entry into circulation or extracellular diffusion into compartments of olfactory nerve bundles leading to entry into the cranial compartment [44]. There is a third pathway where exosomes absorb into the olfactory lymphatics then drain into the cervical lymph nodes of the neck [44]; however, since this is a drainage, this seems to be a clearance pathway and does not seem to deliver the exosomes directly into the brain, which is a possible explanation as to why some exosomes do not reach the brain. Once the exosomes enter the central nervous system (CNS) space either through the olfactory/trigeminal nerve or by paracellular/transcellular transport, exosomes then infiltrate the brain, and an in vivo quantitative analysis study confirms that by showing that ~98% of the intranasally administered EVs incorporate into the cell bodies of neurons in the forebrain, midbrain, and hindbrain in mice [45].

#### 3.1.2. Molecular Mechanisms of Exosome Efficacy

There are multiple mechanisms by which exosomes exert their effect on the brain. One indirect mechanism is soluble and juxtacrine signaling, which does not involve the exosome entering the brain; instead, ligands and receptors on the outer membrane of the exosome bind to specific receptors on the recipient cell membrane like TNFR, Fas, DR4/5, and PD-1, which leads to the activation of intracellular signaling pathways resulting in a cellular response [46]. A more direct uptake mechanism is phagocytosis, which requires FcR and complement receptors found on the outer membrane of the recipient cell [46]. The exosome binds to FcR and the complement receptor via C3/4/5 and IgM, which are ligands that can be found on the outside of the exosome membrane. This binding activates the cell to then uptake the exosome [46]. This is similar to receptor-/raft-mediated endocytosis but with different receptors and ligands such as MFGE8, ICAM1, and integrin [46]. Exosomes can also enter the brain without the need for ligands and receptors through fusion and macropinocytosis [46]. Fusion involves the exosome simply binding and merging with the outer membrane of the recipient cell to release its contents into the cell [46], while macropinocytosis involves the recipient cell forming large protrusions from its outer membrane which wrap and engulf the surrounding solutes [46,47].

The second possible explanation for the greater efficacy of intranasal exosomes compared to intravenous stem cells would be that it is not the route of administration, but rather that it is possible that exosome therapy is inherently superior to stem cell therapy. Stem cells could be inferior to exosomes for varying reasons, one of them being size. Stem cells are much larger than exosomes, with stem cells being ~20–30 µm and exosomes being on the nanoscale [48]. Because of this, stem cells naturally have a more difficult time penetrating certain membranes and tissues to reach their target location. Even when administered intranasally, which intuitively appears to be a more direct route, stem cells seem more inclined to reside in the nares and travel less into the brain, further indicating that stem cells are not as efficient at reaching their target than exosomes regardless of the route of administration. Pre-clinical studies have supported this notion in animal models after intranasal administration (INA) of MSCs. Fluorescence tracking showed that the majority of MSCs tend to not cross the cribriform plate and accumulate in the upper nasal cavity [49]. Furthermore, quantitative data analysis from Danielyan et al. revealed that only ~1000 out of 300,000 administered cells were detected in the brains of mice 1 h post-INA [50].

In terms of comparing efficacy between exosomes and stem cells, it appears that they may be essentially the same. This conceptually makes sense since MSCs can exert their therapeutic effect by secreting EVs that have been shown to mimic the efficacy of MSCs, and exosomes are part of that group of secreted EVs as mentioned before [51,52]. One pre-clinical study compared the therapeutic effects between intravenous MSCs and MSC-derived exosomes in a primary ovarian insufficiency mouse model, and both were able to restore the estrous cycle, serum hormone levels, and fertility [53]. It is important to note that the MSCs did show slightly better efficacy and had a longer effect, but the authors stated that this could be amended by simply increasing the dose of exosomes [53]. Another pre-clinical study performed a similar procedure but in a focal cerebral ischemia mouse model, once again demonstrating that MSCs and MSC-EVs have homogeneous therapeutic outcomes with similar scores in the rotarod test, tightrope test, corner turn test and near indistinguishable neuroprotective effects 28 days post-stroke between the two treatment groups [54]. Despite research indicating that exosomes and stem cells present comparable effects, exosomes could still be seen as better since they cannot self-replicate and potentially mutate [52,55,56]. However, with stem cells having more extensive pre-clinical and clinical data than exosomes, it is difficult to confirm whether exosomes are truly better than stem cells overall, and further comparative research is still required.

##### How Mitochondria Is Affected and Leads to NfL Increase

**Figure 1:**
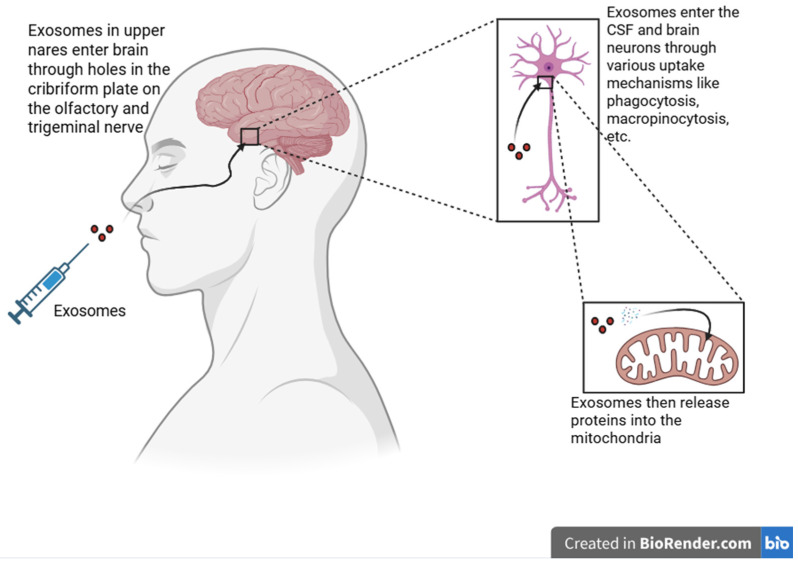
General schematic of the pathway of intranasally instilled exosomes. Created in BioRender. Gerard Pena. (2026) https://app.biorender.com/illustrations/canvas-beta/69a75da1ff4d9a70961ba5f9 (accessed on 28 April 2026).

Another question to consider is by what mechanism the intranasal exosomes improve the clinical picture in these patients with NMND disorders. Studies have shown that mitochondria are affected [57,58,59] in these disorders. Other studies have shown that exosomes burrow through the cell membrane into the cytoplasm of neurons and then release proteins and micro-RNA (miRNA) into the mitochondria as seen in the schematic in Figure 1 [60]. They have been found to decrease mitochondrial apoptosis by regulating the mitochondrial membrane potential stability, reducing mitochondrial stress signaling and delivering targeted miRNA that suppress mitochondrial-dependent apoptotic pathways [61,62,63].

This mechanism is significant because there is an argument to be made that ALS and other NMND disorders are mitochondrial diseases, so treating mitochondria directly may be the key to NMND disease-modifying treatments. There are many studies that suggest that mitochondrial dysfunction causes the progression of NMND disorders. In ALS, mutant SOD1 was found to be associated with the impairment of mitochondrial function [64]. This causes excessive reactive oxygen species production, abnormal protein processing, a reduction in adenosine triphosphate (ATP) production, and even the impairment of complex I in respiratory chain activity in Alzheimer’s [65,66,67]. Since motor neurons have high energy requirements, these factors will inevitably lead to motor neuron death in ALS and other NMNDs [59,68].

When neurons die due to mitochondrial dysfunction, they burst and release neurofilaments into the cerebrospinal fluid (CSF) and bloodstream, which are proteins that are primarily found in neurons and are a key part in the cytoskeletal structure [69,70]. They help maintain the shape of neurons, but they also regulate axoplasmic transport in addition to modulating the speed of nerve signaling in nerve fibers, making neurofilaments crucial in proper neural functions [70]. Among these neurofilaments is the main component, neurofilament light chain (NfL) [70]. NfL is a known biomarker for ALS and neurodegeneration [69,70,71], and since it is released into the CSF and serum, NfL levels can be measured using either a spinal tap or blood test to observe its concentration. Since NfL is released during neuronal damage, a higher NfL level usually indicates a higher level of neurodegeneration and disease progression [69,70,71]. So far in our ongoing, unpublished research, we have seen that serum NfL levels tend to decrease after exosome treatments in ALS patients, which would make sense since, as mentioned before, exosomes can decrease mitochondrial apoptosis [61,62,63], leading to neuron survival and less NfL being released; this is further evidence that treating mitochondria is one of the major mechanisms of exosomes, and it may well be that exosomes decrease cell death by other unelucidated mechanisms.

##### The Role of MicroRNA

As mentioned before, exosomes release proteins and miRNAs into mitochondria when administered. MiRNAs are short non-coding ribonucleic acids (RNAs) that have shown therapeutic potential in various cases [72]. Secretomes contain miRNA but also contain growth factors such as transforming growth factor beta (TGF-β) and anti-inflammatory cytokines such as interleukin-10 (IL-10) that are likely also effective in achieving therapeutic effects [73]. Exosomes are rich in miRNA but have lower concentrations of growth factors and anti-inflammatory cytokines compared to the secretome [74]. It is an area of active research as to whether and to what degree clinical effects are the result of growth factors, anti-inflammatory cytokines or miRNA in these substances. However, clinical trials with exosomes are nearly non-existent, so the relative benefits of miRNA, growth factors, and cytokines in humans remains largely theoretical at this point. At our institute, preliminary results unexpectedly have indicated that secretomes may be more effective than pure miRNA products, indicating a greater role for growth factors and cytokines than previously thought [75,76,77]. The prevailing theory has been that miRNA is the more important agent, but in our studies, this has, so far, not been born out.

Nonetheless, proteomic analysis studies have been conducted to provide a clearer picture of the composition of MSC-derived exosomes, as it has been proposed that the types of miRNAs found in exosomes could affect efficacy, in essence, whether miRNA is more or less important than cytokines and growth factors. It is again an area of active research to determine which types of miRNAs are most effective for various therapeutic applications. The most expressed MSC-derived exosome miRNAs include miR-133, miR-138, miR-124, miR-30, and miR-21, all of which are involved in neuroprotection, neuron growth, and recovery making their use ideal for treating neurodegenerative diseases [78]. While miRNA biology is not yet fully understood, the neural benefits of MSC-derived exosome miRNAs are verified through miRNA administration/overexpression studies to observe the isolated effects of a specific miRNA inhibition to determine what happens when a miRNA is gone and cellular signaling pathway-focused studies to see what role a miRNA has in a specific pathway [78].

##### Exosomes Potentially Affect the Vagus Nerve

The vagus nerve originates in the medulla oblongata [79] which is penetrated by intra-nasally administered substances. It therefore follows that such treatment would have a ready path to stimulate the vagus nerve. The vagus nerve is the main component of the parasympathetic nervous system and is responsible for sending signals from the brain to major organs such as the heart, lungs, and GI tract while also maintaining homeostasis within the body [80]. The vagus nerve also plays a prominent role in inflammation modulation and immune response [80]. Due to this ability, research has been conducted looking at electrical vagus nerve stimulation (VNS) to help treat neuroinflammation in NMND disorders [81]; however, clinical outcomes are not consistent [80]. Currently, there are no studies that look at how administered exosomes may affect the vagus nerve and its activity. To determine the effectiveness of vagus nerve activity, three parameters can be measured via electrocardiogram (ECG): parasympathetic nervous system (PNS) index, sympathetic nervous system (SNS) index, and root mean square of successive differences between normal heartbeats (rMSSD) [82,83]. The PNS index is measured in standard deviations and represents the parasympathetic cardiac activity; typically, positive PNS index values are desirable since it means that the body can recover better in high stress situations [82]. The SNS index is also measured in standard deviations and represents sympathetic cardiac activity, and a negative value is typically desirable since it means that the body is not experiencing an intense stress response [82]. rMSSD is measured in milliseconds and is a parameter of the PNS index that represents heart rate variability, and a higher value is desirable since it represents higher parasympathetic activity [82].

Knowing this, our ongoing research recently revealed that, in 14 patients, there appeared to be a correlation between clinical outcomes after intranasal exosome treatment and vagus nerve activity indicated by changes in SNS index, PNS index, and rMSSD observed before treatment and then after treatment, all of which can be seen in Table 2. Patients who reported better clinical outcomes generally saw an overall improvement in vagus nerve activity. Some patients who did not show a response to treatment did not see a significant change in their vagus nerve parameters. While this area of research is still in the early stages, this promising data shows that stimulating the vagus nerve could be one of the mechanisms of action of exosomes, in addition to mitochondrial protection. Further research focusing on the vagus nerve would need to be carried out, however, to assess this potential correlation. This is the first study to identify a causal connection between vagus nerve activity and the effects of exosomes.

### 3.2. Closing Remarks

The state of the art regarding molecular mechanisms is still primitive, and all molecular mechanisms that have been discussed are all speculative based on what is out there in the current literature. Indeed, most of these products do not have complete proteomic analysis and there is certainly no detailed analysis for miRNA. These products were largely developed for cosmetic purposes for the skin; however, the data from our institute showing groundbreaking effects for neurocognitive disorders shows that their utility is far greater for serious human disease than previously realized [75,76,77]. It is thus imperative that products be characterized in detail and then that their composition is compared to relative clinical efficacy in trials. We are carrying out this work at our institute and hope soon to have better information on what components best correlate with efficacy. And beyond the efficacy of these products for neurologic disorders, there are huge, untapped vistas for other very important clinical applications, e.g., for Alzheimer’s disease and retinal disorders where efficacy has so far not been seen. We believe that with intense research and development it is likely that efficacy will be seen for these disorders and others as well.

## 4. Materials and Methods

A total of 51 patients with ALS, 29 patients with PD, and 6 patients with LBD were enrolled in this study. We instilled secretome and exosome formulations from several different manufacturers into these patients. These secretome and exosome products were derived from cultured MSCs from donated umbilical cord blood. All products have been subject to rigorous testing and analysis by the companies that produce them.

*Inclusion Criteria:* Patients have an established NMND diagnosis by a licensed medical doctor, and the ability to provide informed consent.

*Exclusion Criteria:* Pregnancy, active malignancy, or organ system failure, rendering participation dangerous.

*Treatment Protocol*: Each patient was asked to lie in the supine position. A syringe was used to draw up 4 cc of exosome/secretome solution and was instilled into each nostril in 0.7 cc increments going back and forth between nostrils; small amounts were used to facilitate immediate absorption. This process takes 10–12 min. After instillation, the patient was asked to stay in the supine position for an additional 5 min before they were free to leave the clinic. Patients were treated the consecutive day using the same procedure. Most patients only received secretome; however, patients who did not respond to secretome treatment initially were instead given exosomes. Similarly, if patients were given exosomes at first and had no response, they were given secretome.

*Ratings/Post-Treatment Study Design:* ALS patients were evaluated with the ALSFRS-R, and PD patients were evaluated with the PDQ-39. All patients were evaluated with global improvement ratings during patient follow-up to assess overall well-being. It should be noted that LBD patients were only evaluated with global improvement, as there is no validated rating scale for the disease. Patients were evaluated within 1 month prior to the study to obtain a baseline, and further evaluated one week, one month, 2 months, 3 months, and 6 months post-treatment. All data was applied in a customized spreadsheet.

## Figures and Tables

**Table 1:** Mean ALSFRS-R and PDQ-39 scores.

Follow-Up Time	Mean ALSFRS-R Score	Mean PDQ-39 Score
Baseline	32.92 (n = 48)	25.51 (n = 40)
1 Week	34.05 (n = 39)	21.86 (n = 12)
1 Month	32.45 (n = 41)	24.59 (n = 12)
2 Months	32.80 (n = 32)	20.07 (n = 8)
3 Months	33.97 (n = 29)	21.24 (n = 16)
6 Months	31.10 (n = 10)	22.54 (n = 10)

**Table 2:** Vagus nerve parameters recorded before and after intranasal exosome treatment.

Patient Diagnoses	Pre-tx SNS Index (sd)	Post-tx SNS Index (sd)	Pre-tx PNS Index (sd)	Post-tx PNS Index (sd)	Pre-tx rMSSD (ms)	Post-tx rMSSD (ms)	# of Parameters Improved in	Patient Reported Outcome
ALS #1	3.23	3.06	−2.09	−2.09	27.5	28.2	2/3	Better
ALS #2	3.47	3.49	−1.88	−1.56	11.7	16.7	2/3	No Change
ALS #3	2.29	1.33	−1.32	−0.51	10.3	11.2	3/3	Better
ALS #4	−0.65	−0.97	0.55	1.28	48.4	60.5	3/3	Better
ALS #5	3.01	0.85	−1.95	−1.07	16.5	20	3/3	No Change
ALS #6	8.05	7.61	−2.47	−2.72	5.2	2.9	1/3	No Change
ALS #7	15.15	12.84	−3.07	−3.22	1.8	2	2/3	No Change
ALS #8	4.32	1.22	−1.53	−0.93	10.7	18.5	3/3	Better
Congenital Myasthenic Syndrome	5.36	2.96	−2.98	−2.37	4.8	14.7	3/3	Better
Essential tremor	0.99	1.14	−0.23	−0.06	29.2	28.8	1/3	Better
General health	−1.22	−1.01	1.09	1.17	57.8	60.3	2/3	Better
Trigeminal Neuralgia	0.75	0.13	−0.67	−0.41	36	40.5	3/3	Better
Headaches	0.27	1.12	−0.53	−0.87	33.8	24.8	0/3	No Change
Traumatic Brain Injury	−0.61	−1.09	0.22	0.97	44.6	57.8	3/3	Better

## Data Availability

The original contributions presented in this study are included in the article. Further inquiries can be directed to the corresponding author.

## Abbreviations

The following abbreviations are used in this manuscript:

NMND	Neuromuscular and neurodegenerative
ALS	Amyotrophic lateral sclerosis
LBD	Lewy body dementia
PD	Parkinson’s disease
MSC	Mesenchymal stem cells
EV	Extracellular vesicle
ALSFRS-R	Amyotrophic lateral sclerosis functional rating scale-revised
PDQ-39	Parkinson’s disease questionnaire-39
BBB	Blood-brain barrier
INA	Intranasal administration
CNS	Central nervous system
miRNA	Micro Ribonucleic Acid
ATP	Adenosine triphosphate
CSF	Cerebrospinal fluid
NfL	Neurofilament light chain
VNS	Vagus nerve stimulation
ECG	Electrocardiogram
SNS	Sympathetic nervous system
PNS	Parasympathetic nervous system
rMSSD	Root mean square of successive differences
